# Oxidative stress and endoplasmic reticulum stress in acute kidney injury: mechanistic crosstalk and therapeutic modulation

**DOI:** 10.3389/fmed.2026.1808254

**Published:** 2026-05-01

**Authors:** Xiaoqi Xu, Qiongqiong Xing, Hanqing Li, Zichao Ding, Xianqing Ren

**Affiliations:** 1 Pediatric Medical College, Henan University of Chinese Medicine, Zhengzhou, Henan, China; 2 The First Affiliated Hospital of Henan University of Chinese Medicine, Zhengzhou, Henan, China

**Keywords:** acute kidney injury, apoptosis, autophagy, endoplasmic reticulum stress, oxidative stress

## Abstract

Acute kidney injury (AKI) is a complex syndrome with multiple causes, associated with high morbidity and mortality rates. Despite advances in intensive care, effective therapeutic strategies for AKI remain limited. In this review, we examine the roles of oxidative stress and endoplasmic reticulum stress (ERS) in the pathogenesis of AKI. Oxidative stress, marked by the excessive production of reactive oxygen species (ROS), can trigger ERS, leading to misfolded protein accumulation and activation of the unfolded protein response (UPR) in an attempt to restore cellular homeostasis. When ERS becomes prolonged or excessive, persistent activation of UPR pathways such as IRE1, PERK and ATF6 induces apoptosis and further worsens kidney injury. In addition to apoptosis, oxidative stress and ERS also regulate autophagy, a cellular stress response. Together, these pathways promote cellular dysfunction and advance AKI progression. We also discuss potential therapies that target oxidative stress and ERS, such as antioxidants and pharmacological agents targeting UPR pathways, which may offer promising approaches to mitigate AKI. A deeper understanding of the interplay between oxidative stress and ERS in AKI is essential for developing effective therapeutic interventions to improve patient outcomes.

## 1 Introduction

Acute kidney injury (AKI) is a complex syndrome with multiple causes. Recent statistics show that the incidence of AKI in general hospitalized patients is approximately 10–15%, while it can exceed 50% in intensive care units (1). A global review covering the past 15 years reports an annual incidence of 114–174 per 100,000 people, with an estimated total of 13.3 million cases in 2017 (2). The burden of disease is particularly significant in low- and middle-income countries due to factors such as endemic diseases, water pollution, and socio-cultural influences (1). Mounting evidence also shows that AKI significantly increases the risk of both short- and long-term complications, affecting multiple organs, and raises healthcare costs (3). Clinically, ischemia–reperfusion injury (IRI), sepsis, and nephrotoxic substances are the primary causes of AKI. Given the strong association between AKI and poor prognosis with high mortality, and the limited effective treatment options currently available, there is an urgent need to elucidate the pathogenesis of AKI and identify potential therapeutic interventions.

In most cases, the integrity of renal parenchyma is compromised by renal tubular dysfunction caused by low perfusion or by direct damage to renal interstitial tissues and cellular functions by “toxins” (4). Oxidative stress triggers a cascade of reactions by generating reactive oxygen species (ROS) and their metabolites. These products can act as ligands for various receptors, such as Toll-like receptors, which in turn activate “alarms” that perpetuate the harmful processes in AKI. These “toxins” in circulation are inflammatory products that mediate the extension of damage and hemodynamic imbalance (5). Critical illness intertwines with acute inflammation and the subsequent production of ROS, further exacerbating oxidative stress responses. ROS spread AKI through gap junctions formed by connexin 32 (Cx32) (6). The activation of the ROS-ERS-apoptosis signaling pathway plays a significant role in I/R-induced AKI, and both ROS elimination and ERS inhibition can prevent I/R-induced AKI (6). Recent evidence further indicates that ISL1-overexpressing bone marrow mesenchymal stem cells attenuate oxidative stress, inflammation, and tubular apoptosis in renal ischemia–reperfusion injury through paracrine actions, highlighting the therapeutic potential of targeting oxidative stress-related injury pathways in AKI (7). ROS can promote ERS, which subsequently contributes to renal tubular cell apoptosis (8). This suggests that oxidative stress may promote AKI through ERS. Endoplasmic reticulum stress (ERS) plays a crucial role in the progression of AKI. Under pathological conditions that increase protein folding load or disrupt normal folding processes, misfolded proteins accumulate in the ER, leading to ER expansion and triggering ERS, thereby activating the unfolded protein response (UPR). Studies have shown that disruption of ER protein homeostasis and dysfunction of the UPR promote the onset and progression of kidney diseases (9). The primary UPR pathways activated by ERS include IRE1α, PERK, and ATF6, all of which are involved in apoptosis and autophagy, ultimately leading to cell death. In addition, regulated cell death pathways, including ferroptosis (10), pyroptosis (11) and NETosis (12), also contribute substantially to the pathogenesis of AKI, and recent reviews summarizing therapeutic inhibitors that target these processes are available for further reference.

This review examines the sources of oxidative stress in AKI, the mechanisms by which oxidative stress-induced ER stress contributes to cell death through autophagy and apoptosis, and discusses the potential of antioxidants to alleviate these processes in the treatment of AKI.

## 2 Methods

This review was conducted as a narrative literature review to summarize the current evidence on the interplay between oxidative stress and endoplasmic reticulum stress in acute kidney injury. Literature retrieval was performed in the PubMed database from database inception to January 2026. Based on the scope and structure of this review, both Medical Subject Headings and free-text terms were used. The main search terms included acute kidney injury, AKI, renal ischemia–reperfusion injury, sepsis-associated acute kidney injury, nephrotoxic acute kidney injury, oxidative stress, reactive oxygen species, ROS, mitochondrial dysfunction, mitochondrial ROS, NADPH oxidase, NOX, endoplasmic reticulum stress, ER stress, ERS, unfolded protein response, UPR, IRE1, PERK, ATF6, apoptosis, autophagy, ferroptosis, antioxidants, and therapeutic targets. Boolean operators such as AND and OR were used to combine search terms as appropriate in order to improve retrieval sensitivity and relevance.

The inclusion criteria were as follows: studies directly relevant to the topic of this review, with a focus on oxidative stress, endoplasmic reticulum stress, and their crosstalk in AKI. Studies addressing mechanisms of AKI pathogenesis, cell fate regulation, or potential therapeutic interventions, including but not limited to apoptosis, autophagy, ferroptosis, and antioxidant- or UPR-targeted therapies. Article types including basic experimental studies, clinical studies, and high-quality reviews and priority given to recent English-language publications with clear mechanistic insights and high relevance to the aims of this review.

The exclusion criteria were as follows: (1) Studies not directly related to AKI or the oxidative stress-ER stress axis. (2) Duplicated publications, studies with limited information, or low-evidence-value reports. (3) Conference abstracts, editorials, letters, and articles without accessible full text. The eligible studies were then screened, organized, and synthesized to construct the framework of this review.

### 2.1 Oxidative stress: sources and consequences in AKI

Under physiological conditions, the kidneys generate ROS, such as superoxide anion (O₂^−^), hydrogen peroxide (H₂O₂), peroxynitrite (ONOO^−^), and hydroxyl radicals (·OH). These ROS are efficiently cleared by enzymatic systems, for example superoxide dismutase (SOD), catalase, and glutathione peroxidase (GPX), as well as non-enzymatic systems, including glutathione, vitamins C, and E (13). However, ROS production must be kept at a baseline, non-toxic level, a balance maintained mainly by intracellular antioxidants. Any deviation from this delicate balance, such as increased ROS generation or diminished free radical clearance, disrupts redox homeostasis and triggers oxidative stress (14). When the production of ROS exceeds the capacity of the antioxidant defense system, oxidative stress ensues, typically resulting in tissue damage (15).

#### 2.1.1 Mitochondrial dysfunction and mtROS surge

The kidneys have high energy demands as they perform essential functions, including the clearance of metabolic waste, reabsorption of nutrients, regulation of electrolyte balance, maintenance of acid–base homeostasis, and blood pressure control. The ATP required for these processes is derived from oxidative phosphorylation (OXPHOS) and anaerobic glycolysis (16). Studies show that during the early stages of AKI, ATP levels decrease and mitochondrial structure is altered, leading to energy metabolism dysregulation (17). The number of mitochondria in the kidneys varies depending on the cell type, which results in differing levels of ROS production across different renal structures (18). Mitochondrial ROS (mtROS) arise at complexes I and III of the electron transport chain (ETC) when oxygen accepts electrons from nicotinamide adenine dinucleotide (NADH) or flavin adenine dinucleotide (FADH₂) (19). Under normal respiratory conditions, mtROS are generated because of proton leakage; however, their production increases significantly und ERS conditions (20). In AKI, the surge in ROS can directly oxidize and damage mitochondrial lipids and proteins, impairing ETC function and increasing mitochondrial membrane permeability, thereby disrupting mitochondrial bioenergetics (21). In addition, in proton pump inhibitor-associated kidney injury, ROS derived from mitochondria and NADPH oxidase can drive tubular cell death by promoting lipid peroxidation, mitochondrial membrane potential loss, and permeability transition, while acting synergistically with ATP depletion and lysosomal dysfunction (22). In AKI rat models, changes in mitochondrial NADH levels, proton motive force, elevated O₂ levels, and mitochondrial structural fragmentation have been observed, highlighting the key role of mitochondrial dysfunction in AKI pathogenesis (23). Furthermore, renal ischemia has been reported to downregulate peroxisome proliferator-activated receptor gamma (PPARγ), leading to suppressed UCP1 expression, which promotes mtROS production and induces oxidative stress and AKI (24). UCP1, located in renal tubular epithelial cells, is downregulated in a time-dependent manner during renal ischemia–reperfusion, and its absence amplifies oxidative stress levels and exacerbates ischemic AKI in mice (24). Studies indicate that during ischemia or hypoxia, the opening of mitochondrial permeability transition pores (MPTP), downregulation of NAD^+^ synthesis-regulating factors as NAMPT, and overactivation of poly (ADP-ribose) polymerase-1 (PARP-1) lead to decreased levels of sirtuins, SIRT1 and SIRT3 (25). Sirtuins, an important family of regulatory proteins involved in cellular metabolism, are NAD^+^ dependent enzymes that regulate antioxidant defense and oxidative stress through DNA repair and deacetylation, with SIRT1 located in the nucleus and SIRT3 in mitochondria (26). Loss of SIRT1’s de-acetylase activity allows its substrates to remain acetylated, which switches off PPARGC1α, dampens manganese superoxide dismutase (MnSOD) activity, boosts ROS production and suppresses mitochondrial fatty-acid *β*-oxidation, all of which intensify oxidative stress (25). Likewise, SIRT3 deficiency undermines mitochondrial architecture, ATP output and antioxidant capacity, thereby heightening oxidative stress, inflammation and apoptosis (26). Comparative metabolomics points to reverse electron transport (RET, a mitochondrial process in which electrons flow in the reverse direction through the electron transport chain, leading to ROS production at complex I) through complex I of the electron-transport chain (ETC) as a key generator of superoxide (O₂•−) (27). Normally, oxidative phosphorylation is tightly regulated, with only ~1–2% of electrons leaking to form O₂•− (28). Complexes I and III are the chief sources of this leak, and in endothelial cells complex III predominates (29, 30). Once formed, matrix O₂• − is rapidly converted by MnSOD to H₂O₂, which can diffuse across the outer mitochondrial membrane into the cytosol, where it influences multiple targets (31)—for example, activating redox-sensitive transcription factors, pro-inflammatory cytokines and inflammasomes (32). Mitochondrial ROS can also interact with nitric-oxide synthase, further amplifying oxidative-stress signaling (33).

#### 2.1.2 NADPH oxidase-driven renal oxidative burst

NADPH oxidase (NOX) enzymes serve as the dominant renal sources of reactive oxygen species. Of the seven known isoforms, NOX1, NOX2, NOX4 and NOX5 are the most abundantly expressed in kidney cells (34). Superoxide (O₂•−) generation by NOX1 and NOX2 depends on the assembly of the membrane partner p22-phox with the cytosolic subunits p47-phox, p67-phox and the small GTP-binding protein Rac1 (35). In contrast, NOX4—highly enriched in mitochondrial membranes of renal cells—bypasses these cytosolic partners and produces hydrogen peroxide (H₂O₂) directly (36). In AKI and chronic kidney disease (CKD) models, elevated levels of NOX isoforms lead to excessive ROS production (37). The excess ROS produced by NOXs (in the renal cortex, proximal and distal tubules) is associated with enhanced mtROS, creating a pathological cycle of ROS generation that promotes the transition from AKI to CKD (38). In partial nephrectomy models, mtROS can also activate NOXs, increasing inflammation and fibrosis (39).

In renal disorders, excessive mitochondrial ROS together with ROS derived from NOX enzymes intensify mitochondrial injury and dissipate the organelle’s membrane potential (40, 41). In diabetic nephropathy, hyperglycaemia-driven advanced glycation end-products (AGEs) bind their receptor for AGEs (RAGE) and activate NOX isoforms, boosting ROS production (42). Protein kinase C-*ε* amplifies this effect by phosphorylating the organizer subunit p47-phox, thereby further stimulating NOX activity (43). The resulting bursts of ROS open mitochondrial ATP-sensitive K^+^ (mt-K_ATP) channels, precipitating a drop in mitochondrial membrane potential (44). In AKI models, NOX inhibitors such as apocynin reduce mtROS generation (45, 46), supporting the interaction between NOXs and mitochondria. Angiotensin II (AngII) and AGEs bind to their receptor, activating TGFβ1 and the NF-κB redox-sensitive signaling pathway (47). mtROS also promote the activation and nuclear translocation of TGFβ1 through upregulation of Smad2/3; mitochondrial-targeted antioxidants, such as mitoQ and mitoTEMPO, can block this process (48). mtROS activate NF-κB by promoting monocyte/macrophage infiltration, leading to interstitial inflammation, and antioxidants like curcumin can inhibit this effect (49). mtROS can also activate NF-κB in macrophages by altering the disulfide bond of IKKγ, independent of NOX2 products (50). Both TGFβ1 and NF-κB localize to mitochondria, regulating mitochondrial proteins (51). In DN, high glucose induces Smad4 translocation to mitochondria, reducing OXPHOS, leading to inflammation, fibrosis, and podocyte damage (51). The ROS derived from mitochondria and NOX enzymes can also damage phospholipids and mtDNA, which lacks histone protection and is particularly sensitive to ROS, leading to loss of integrity and accumulation of mutations (52). mtROS also promote the opening of the MPTP to the cytoplasm (40), while oxidizing cardiolipin and other phospholipids, causing mitochondrial membrane depolarization, MPTP opening, and decreased ETC activity (53). Thus, the pathological interaction between NOX enzymes and mitochondria promotes ROS generation, disrupts mitochondrial metabolism and the integrity of biomolecules, and exacerbates mitochondrial dysfunction.

#### 2.1.3 Immune-cell-derived ROS in sepsis-associated AKI

Sepsis is the leading cause of acute kidney injury (AKI) in the intensive-care setting, responsible for roughly 40–50% of all episodes and markedly raising in-hospital mortality risk (54). In septic AKI (SAKI), intrarenal capillary perfusion around tubular epithelial cells decreases, while oxidative stress rises (55). These two early insults—microvascular dysfunction and redox imbalance—act synergistically to propel SAKI. Experimental work links sluggish microvascular flow tightly to heightened oxidative stress, showing that capillary hypoperfusion and ROS generation occur together near renal tubules (56). During sepsis, pathogen invasion activates innate-immune sentinels—especially macrophages and neutrophils—which deploy reactive species such as superoxide (O₂•−), hydroxyl radical (•OH), peroxynitrite (ONOO^−^) and hypochlorous acid (HOCl) to kill microbes (57). Myeloperoxidase in neutrophils and macrophages converts H₂O₂ plus Cl^−^ into highly bactericidal hypochlorous acid (58). In SAKI, dendritic cells and neutrophils are pivotal sources of renal injury and ROS (57): dendritic cells amplify oxidative stress indirectly by recruiting/priming neutrophils, while neutrophils destroy pathogens via either phagocytic “oxidative bursts” or NETosis. Both processes rely on ROS—phagocytosis demands rapid ROS generation, whereas NETosis is triggered by NOX2-dependent O₂• − production (59, 60). The complete assembly and activation of NOX2 generate O₂• − by transferring electrons from NADPH to molecular oxygen, and it is reported that activated neutrophils can generate approximately 10 nmol of O₂•^−^ per million cells per minute during the oxidative burst (33). In the SAKI mouse model, inhibiting NOX2 and inducible nitric-oxide-synthase (iNOS) activity reduces renal damage (57). Furthermore, sepsis-related inflammatory responses upregulate iNOS, leading to large amounts of NO production, and excess NO can cause uncoupling of endothelial nitric oxide synthase (eNOS), further generating O₂•^−^ and inducing oxidative stress (61). eNOS generates NO and citrulline through a two-step O₂-dependent arginine monooxygenase reaction. Its electron transfer is strictly regulated, and when eNOS becomes uncoupled, electrons shift to molecular oxygen rather than arginine, producing O₂•^−^ (62). Excess NO can also competitively react with O₂• − to form ONOO^−^, which leads to protein nitration in renal tubules and directly damages tubular cells (59). ONOO^−^ is a potent oxidant, with greater toxicity than NO, and is involved in endothelial dysfunction (63). Rosales et al. reported in a mouse model of renal ischemia–reperfusion injury that oxidative signals were markedly increased in the injured kidney, whereas this phenomenon was abolished in gp91^phox^/NOX2-deficient mice, suggesting that myeloid cell-mediated, NOX2-dependent oxidative burst may contribute to the establishment of the renal oxidative microenvironment in AKI and thereby promote tissue injury progression (64). In parallel, Zhu et al. showed that the Irg1–itaconate/Nrf2 axis exerts a protective effect in AKI. Irg1 deficiency aggravated inflammatory responses and renal injury after ischemia–reperfusion, whereas dimethyl itaconate alleviated renal injury, suppressed macrophage activation and inflammatory cytokine release, and reduced ROS generation as well as apoptosis-related changes in renal cells after hypoxia–reoxygenation. These effects were markedly attenuated when Nrf2 was inhibited, suggesting that this pathway may confer renoprotection by simultaneously restraining immune-cell inflammatory activation and mitigating oxidative stress-mediated injury in renal parenchymal cells (65).

#### 2.1.4 Ferroptosis: iron-catalyzed lipid peroxidation

Ferroptosis—a non-apoptotic, iron-dependent mode of cell death—also fuels ROS production during AKI (66). It is initiated by Fenton chemistry, in which ferrous iron (Fe^2+^) reacts with H₂O₂ to yield highly reactive hydroxyl radicals (•OH) that drive lipid peroxidation (LPO) (67). Excessive oxidation of polyunsaturated fatty acid chains in membrane phospholipids, coupled with inadequate glutathione-peroxidase-4 (GPx4) activity, ultimately ruptures membranes and causes cell death (68). Thus, iron handling and LPO are the two key control points of ferroptosis (69). In the bloodstream, iron circulates as ferric iron (Fe^3+^) bound to transferrin, enters cells via the transferrin receptor, and is reduced to Fe^2+^ inside endosomes. Divalent metal transporter 1 then releases Fe^2+^ into the cytosolic labile-iron pool (70). Within the cell, iron is chiefly stored in ferritin, which contains light (FTL) and heavy (FTH) subunits (71). FTL localizes predominantly to the cytosol, whereas FTH is found in both cytoplasm and nucleus, suggesting additional antioxidant or gene-regulatory roles in the latter compartment (72). Importantly, FTH’s ferroxidase activity converts Fe^2+^ to Fe^3+^ and locks it inside the ferritin shell, thereby limiting the pool of redox-active iron that can drive further ROS formation (73). Ferroptosis is marked by the accumulation of iron and ROS, suppression of system Xc^−^, and reduced GPX4 activity, leading to reduced cysteine uptake, glutathione (GSH) depletion, and the release of molecules like arachidonic acid (74). GSH is a major intracellular antioxidant, and its depletion leads to accumulation of iron-dependent lipid ROS, causing cell death (75). GPX4, a GSH-dependent enzyme, reduces lipid peroxides to alcohols; loss of GPX4 activity likewise induces ferroptosis (76). Activation of Nrf2, which upregulates GPX4, SLC7A11 and HO-1, inhibits ferroptosis in the kidney (77). Recent evidence indicates that the ferroptosis inhibitor Cpd-A1 attenuates renal injury and lipid peroxidation in ischemia–reperfusion- and sepsis-associated AKI models, suggesting that ferroptosis targeting may represent a potential strategy for mitigating oxidative stress-related kidney injury in AKI (78).

#### 2.1.5 Environmental and metal pollutants as oxidative triggers

Although the precise pathways by which metals injure the kidney are incompletely defined, mounting evidence indicate that metal-driven oxidative stress is a central culprit (79). Transition metals catalyze redox reactions involving cellular macromolecules, and this pro-oxidant activity underlies their toxicity in multiple renal compartments—especially the proximal tubules, which bear the brunt of metal assault alongside the glomerulus, vasculature and distal nephron (80). Classic hallmarks of metal-induced nephrotoxicity include depletion of intracellular GSH and other radical scavengers, suppression of antioxidant enzymes, and sharp rises in ROS (81). Different metals promote oxidative stress through distinct but overlapping mechanisms. Mercury, cadmium and nickel directly consume GSH; chromium, iron, copper and vanadium undergo redox cycling; and metals such as iron, chromium, vanadium, copper and cobalt generate O₂• − and •OH via Fenton chemistry. Arsenic, by contrast, is thought to bind critical thiol groups in proteins and antioxidants (82, 83). Cadmium preferentially accumulates in mitochondria, where it stalls complex III, spawns free radicals and activates caspases, culminating in tubular injury (84, 85). Lead magnifies oxidative damage by triggering the mitochondrial permeability transition, uncoupling the respiratory chain and depleting GSH, thereby driving sustained ROS production and worsening renal dysfunction (86).

Agricultural and environmental chemicals can also induce AKI, with the toxicological pathways of many pesticides related to oxidative stress, potentially through their metabolites, mitochondrial damage, or inhibition of cellular antioxidant defense systems (87). These toxins can induce oxidative stress through redox cycling mechanisms: NADPH one-electron reduction generates free radicals, which then transfer electrons to O₂, forming O₂•^−^. When NADPH is depleted, O₂• − undergoes self-reaction to generate OH•, resulting in cell death (88). Mitochondrial dysfunction generates excessive ROS, accumulates cytosolic fatty acids, and promotes increased peroxisomal and endoplasmic reticulum fatty acid oxidation, which also contributes to pesticide-induced AKI (89).

Taken together, oxidative stress in AKI reflects an imbalance between ROS generation and antioxidant defenses. Electron leak and reverse electron transport at complexes I/III drive mtROS surges; reduced SIRT1/3 activity with impaired MnSOD/PPARGC1α exacerbates bioenergetic failure, increases membrane permeability, and engages NF-κB and TGFβ1 signaling. Renal NOX1/2/4/5 provide parallel ROS sources and form a feed-forward loop with mitochondria that accelerates the AKI-to-CKD transition; in sepsis, NOX2/MPO/iNOS-dependent ROS/RNS from immune cells, compounded by microvascular dysfunction, further injure endothelium and tubules. Ferroptosis and exposure to metals/pesticides amplify oxidative injury via Fenton chemistry, glutathione depletion, and GPX4 inhibition. Accordingly, disrupting the NOX–mitochondria loop, preserving mitochondrial homeostasis, and targeting ferroptosis are promising strategies to mitigate oxidative stress and limit disease progression.

### 2.2 Crosstalk between oxidative and endoplasmic reticulum stress in AKI

Studies have shown that high doses of tunicamycin cause widespread damage in the kidneys of mice, accompanied by an increase in oxidative stress (90). Elevated oxidative stress contributes to kidney failure during ERS; high levels of H₂O₂ lower IRE1 levels and inhibit XBP-1 splicing, an effect largely prevented by co-treatment with antioxidants (90). Similarly, research by Zhang et al. demonstrated a significant increase in ROS generation in the kidneys of LPS-induced AKI mice, where the HDAC inhibitor MS-275 attenuated oxidative stress, normalized ER-stress markers (GRP78, caspase-3 and caspase-12) and improved renal function (91). These studies collectively confirm that the increase in ROS in AKI alters ER oxidative stress, thereby influencing cell death. ERS plays a critical role in AKI progression. For example, Huang et al. showed that TNFα knockout mice are more susceptible to cell death induced by ERS, thereby increasing the kidneys’ sensitivity to acute ERS damage (92).

**Figure 1:**
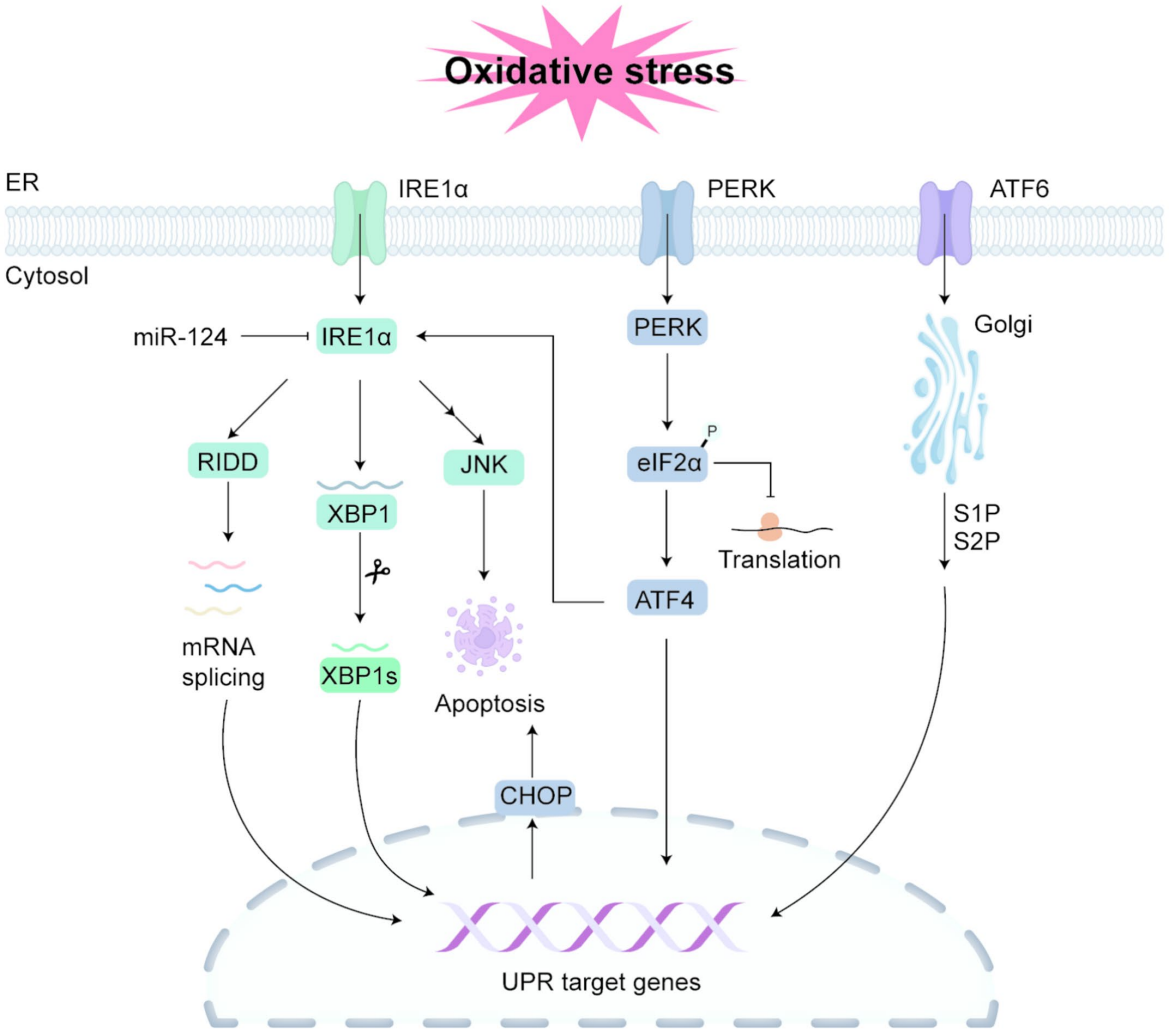
Major pathways of UPR activation under endoplasmic reticulum stress. Endoplasmic reticulum stress (ERS) triggers autophosphorylation of the IRE1α kinase, promoting the splicing of X-box binding protein 1 (XBP1) mRNA to generate the spliced form, XBP1s. XBP1s acts as a transcription factor to activate genes involved in the unfolded protein response (UPR). IRE1α can also degrade various mRNAs through the regulated IRE1-dependent decay (RIDD) pathway to alleviate protein load. In addition, IRE1 may induce renal cell apoptosis via the JNK pathway. MiR-124 can bind to the 3’UTR of IRE1α and downregulate its expression, thereby inhibiting the IRE1/XBP1 pathway and reducing ERS and apoptosis. The PERK pathway is activated through homodimerization and autophosphorylation of its luminal domain. Activated PERK phosphorylates eukaryotic initiation factor 2α (eIF2α), leading to a temporary attenuation of protein synthesis. Phosphorylation of eIF2α also promotes the translation of activating transcription factor 4 (ATF4), which induces the expression of C/EBP homologous protein (CHOP), a key pro-apoptotic transcription factor. Under non-stressed conditions, ATF6 (a type II transmembrane protein) is mainly localized to the ER membrane. Upon ER stress, ATF6 translocates to the Golgi apparatus, where it is sequentially cleaved by Site-1 protease (S1P) and Site-2 protease (S2P). The cleaved active fragment then translocates to the nucleus, where it functions as a transcription factor to initiate the transcription of UPR-related genes, thereby modulating downstream targets of the UPR.

In pathological states where the protein folding load must be increased or normal folding processes are disrupted, misfolded proteins accumulate in the ER, leading to ER swelling and triggering ERS and activation of the UPR (93, 94). The UPR is a typical adaptive stress response, and there is substantial evidence that disturbances in ER protein homeostasis and UPR dysfunction promote the onset and progression of kidney diseases (9). The unfolded-protein response (UPR) is driven by three principal signaling branches (Figure 1), each launched by one of three ER-resident sensors: activating transcription factor 6 (ATF6), inositol-requiring enzyme 1α (IRE1α) and PKR-like ER kinase (PERK) (95). Under basal conditions these sensors are kept inactive through association with the chaperone BiP. When misfolded proteins accumulate, BiP disengages to bind the aberrant polypeptides, freeing the sensors to activate their respective pathways (96).

#### 2.2.1 Unfolded protein response arms in AKI

IRE1α is a highly conserved ER-stress sensor that combines both serine/threonine-kinase and endoribonuclease functions (97). By activating the transcription factor XBP1, IRE1α orchestrates an adaptive program that enhances protein folding and disposal capacity within the ER (98). ERS prompts IRE1α to autophosphorylate, switching on its RNase domain to excise a short intron from X-box-binding protein-1 (XBP1) mRNA and generate two products: the spliced form XBP1s and the unspliced precursor XBP1u (99). XBP1s up-regulates genes that expand ER quality-control and degradation machinery, whereas XBP1u acts as a brake, repressing this transcriptional response (100). Furthermore, IRE1α has a function independent of XBP1 mRNA splicing: it can reduce protein load through the regulated IRE1α-dependent decay (RIDD) pathway, which cleaves multiple mRNAs (101). In nephrotoxic AKI models, IRE1 alleviates oxidative stress and inflammation by reducing the accumulation of misfolded proteins and modulating JNK activation, a key pathway that induces renal cell apoptosis. However, persistent activation of IRE1 may promote pro-inflammatory responses and exacerbate kidney damage. Renal ischemia/reperfusion (I/R) injury is one of the primary causes of acute kidney failure, and during I/R, a large amount of ROS is generated, leading to the accumulation of misfolded and unfolded proteins in the ER, triggering the UPR and activating IRE1α to promote the degradation of misfolded proteins (102). Additionally, the calcium overload and ROS generation during reperfusion exacerbate oxidative stress, further amplifying UPR and apoptosis (103). In renal I/R injury models, the expression of ERS-related proteins such as IRE1α, XBP1, and GRP78 is significantly increased (104), and miR-124 can bind to the 3’UTR of IRE1α, downregulating IRE1α expression and inhibiting the IRE1/XBP1 pathway, reducing ERS and apoptosis, and thus alleviating renal I/R injury (104). In HgCl₂-induced AKI mouse models, upregulation of the PERK/eIF2α branch and activation of the PERK/ATF-4 branch promote the upregulation of ATF-4, ATF-6, and IRE1α, which jointly upregulate GADD-153, leading to the activation of caspase-12 and caspase-3, causing death of renal tubular and glomerular cells (105). Similarly, the use of nephrotoxic drugs such as cisplatin, gentamicin, and paracetamol activates IRE1α and upregulates sXBP1 expression in rats (106). Studies indicate that interventions targeting IRE1 can reduce oxidative damage and inflammation, providing protection against AKI in drug-induced nephrotoxicity models (107).

PERK, a key sensor of ER stress, becomes active when its lumenal domains mediate homodimerization followed by autophosphorylation. Once PERK is activated it phosphorylates the *α*-subunit of eukaryotic initiation factor 2 (eIF2α). This modification transiently stalls global translation, easing the influx of nascent polypeptides into the ER and buying time for proper folding. Paradoxically, the same eIF2α phosphorylation event selectively favors translation of activating transcription factor 4 (ATF4)—a pivotal UPR effector that controls genes governing antioxidant defense, apoptosis, autophagy, and amino-acid metabolism and transport (108, 109). Among the ATF4-driven outputs is the transcription factor CHOP (C/EBP-homologous protein), whose abundance rises markedly in response to PERK-eIF2α signaling and promotes pro-death programs when ERS is unresolved (110). Renal I/R inhibits protein synthesis, and one of the key molecular mechanisms is the phosphorylation of eIF2α. In a rat model of “10 min of cardiac arrest ischemia + 10 min of reperfusion,” renal tissue p-eIF2α levels were approximately 20 times higher than baseline, indicating a sharp “braking” of translation initiation. This increase was accompanied by PERK activation, suggesting that this signaling originates from the ERS pathway (111). Studies have shown that Salubrinal, a selective inhibitor of eIF2α dephosphorylation, increases eIF2α phosphorylation levels. When combined with cisplatin, Salubrinal significantly further enhances eIF2α phosphorylation, triggering ERS-dependent renal tubular cell apoptosis (112). This process, along with enhanced oxidative stress, is mutually promoted, and N-acetylcysteine (NAC) can simultaneously inhibit LPO, reversing the nephrotoxicity of Salubrinal (112).

In resting cells, ATF6 predominantly resides in the ER membrane (113). When the ER is stressed, ATF6 is translocated to the Golgi apparatus, where it is cleaved sequentially by Site-1 protease (S1P) and Site-2 metalloprotease (S2P) (114). The cleaved active fragment then enters the nucleus, acting as a transcription factor to initiate the transcription of UPR-related genes, thereby regulating the expression of downstream UPR target genes (99). Studies have shown that the ATF6-UPR branch is significantly activated in human AKI pathological specimens and AKI mouse models, manifested by increased ATF6 nuclear translocation and upregulation of downstream target proteins (GRP78/BiP and CHOP), suggesting that ATF6 participates in renal stress-induced damage (115). Histone deacetylase 6 (HDAC6) inhibitor CAY10603 can suppress ATF6 activation and exhibit improvement in AKI (115).

#### 2.2.2 Sex- and age-dependent modulation of the oxidative stress–ERS axis in AKI

Preclinical evidence indicates that activation the activation of the oxidative stress–ERS axis in AKI is modulated by individual factors such as sex and age (116). In male mice, tunicamycin elicits more pronounced upregulation of GRP78–XBP1s–CHOP and Bax/Caspase-3–mediated apoptosis than in females, which are inherently more resistant; exogenous testosterone abolishes this female advantage, consistent with androgens amplifying ER-stress toxicity in rodents (116). In I/R models, female kidneys depend on estrogen–SIRT3 signaling to maintain mitochondrial fusion and suppress ROS bursts, thereby attenuating downstream ER stress; silencing Sirt3 negates this protection, while relevance to humans remains to be determined (117, 118). Male spontaneously hypertensive rats display sustained 12/15-lipoxygenase activation seven days post-ischemia, resulting in elevated ROS–ERS burden and delayed functional recovery, indicative of a sex-biased lipoxygenase–ROS–ERS feedback loop (119). Aging further exacerbates vulnerability: in aged or overweight mice, reduced basal NRF2 antioxidant capacity and diminished mitochondrial reserve in aged or overweight mice lead to exaggerated ROS surges and excessive UPR activation during I/R (120), whereas short-term fasting reinduces NRF2, lessens acute necrosis, and mitigates BUN elevation in these models, consistent with metabolic reprogramming as a protective mechanism; kidney-specific causal links in humans remain to be established (121). Finally, in aged female mice, the sulfide donor diallyl-trisulfide significantly ameliorates I/R injury by downregulating p-PERK, p-IRE1α, and ATF6, while improving renal perfusion and angiogenesis in this model; broader applicability across sex and species requires further study (122).

#### 2.2.3 ERS-mediated cell-fate decisions

ERS plays a “double-edged sword” role in determining cell fate: its impact can both protect and harm the cell, depending on the intensity and duration of the stress. Under moderate stress conditions, ERS can induce autophagy to degrade abnormal proteins, maintain homeostasis, and improve cell survival (123–125). However, persistent ERS leads to sustained increases in intracellular Ca^2+^ levels and long-term activation of the UPR, leading to mitochondrial dysfunction and triggering apoptosis to eliminate severely damaged cells (126). Thus, the early stage of the UPR tends to promote cellular adaptation, but if the stress continues, it shifts toward inducing apoptosis (127, 128).

##### 2.2.3.1 CHOP-dominant apoptotic signaling

Apoptosis is the best-characterized form of programmed cell death, relying on a caspase cascade for execution (129). A defining event is the release of mitochondrial cytochrome c, governed by the interplay of pro- and anti-apoptotic BCL-2-family proteins and mediated by initiator caspases (−8, −9, −10) and downstream effector caspases (−3, −6, −7) (130, 131). Two broad routes feed into this process: an extrinsic pathway launched at cell-surface death receptors, and an intrinsic pathway that comprises both mitochondrial and ER branches. Cellular stressors—such as toxins or DNA damage—activate BH3-only proteins, prompting BAX/BAK oligomerisation, MOMP, cytochrome c release and caspase activation (132, 133). Within the ER limb, caspase-12—an ER-resident cysteine protease—becomes selectively activated, then engages caspase-9 and caspase-3 to drive apoptosis (134).

**Figure 2:**
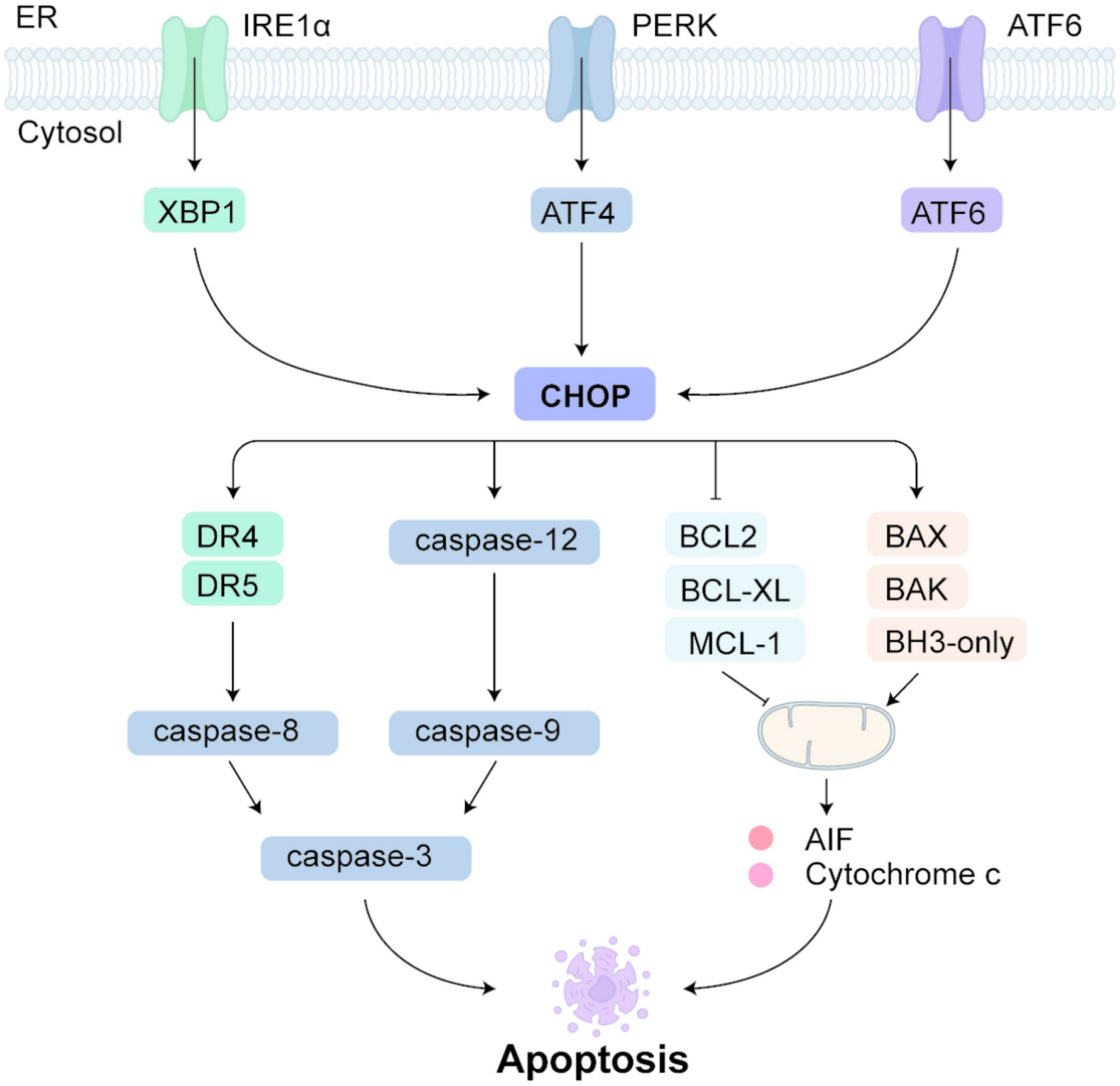
ER stress and apoptosis. In the ER stress-induced apoptotic pathway, ATF6, IRE1, and ATF4 bind and activate the CHOP promoter under ERS conditions. CHOP activation promotes the translocation of caspase-12 into the cytoplasm, initiating a caspase cascade that leads to ERS-dependent apoptosis. CHOP also upregulates the expression of death receptors DR4 and DR5, activating caspase-8 and promoting cell death via the death receptor pathway. In addition, CHOP suppresses the anti-apoptotic expression of BCL2, BCL-XL, and MCL-1, while promoting the expression of pro-apoptotic proteins BAX, BAK, and BH3-only proteins. This facilitates mitochondrial outer membrane permeabilization (MOMP), resulting in the release of cytochrome c and apoptosis-inducing factor (AIF), ultimately leading to programmed cell death.

Under severe ERS, all three unfolded-protein-response sensors (ATF6, IRE1 and the PERK-dependent ATF4) converge on the CHOP promoter (Figure 2) (135, 136). CHOP then propels apoptosis by translocating caspase-12 to the cytosol and initiating the downstream caspase cascade (137). It can do so through both extrinsic and intrinsic routes: CHOP boosts the death-receptor proteins DR4 and DR5, leading to caspase-8 activation, while simultaneously modulating BCL-2-family gene expression (132, 138, 139). Specifically, CHOP suppresses the anti-apoptotic members BCL-2, BCL-XL and MCL-1 and elevates the BH3-only protein BIM, thereby favoring BAX and BAK activation (134, 140–143). Subsequent mitochondrial permeabilisation unleashes cytochrome c and apoptosis-inducing factor (AIF), sealing the apoptotic fate of the cell (144).

In a Sprague–Dawley rat IRI model, excessive ERS increases CHOP levels, activating caspase-3 and BAX, and inducing apoptosis (145). In another study using live rats and NRK-52E cell models, IRI significantly upregulated ERS response proteins GRP78, XBP1, and ATF6. After IRI, BAX and cleaved caspase-3 levels significantly increased, while BCL2 expression markedly decreased (146). In AKI mouse kidney tubule cells, PERK and its downstream protein translation regulator, eIF2α, showed transient phosphorylation (111). CHOP, the pro-death effector downstream of PERK, has been shown to worsen apoptosis and renal dysfunction in murine models of ischaemia–reperfusion injury (IRI), toxin-mediated AKI and obstructive nephropathy, underscoring the central role of the PERK–CHOP axis in kidney damage (147, 148). A similar pattern emerges in sepsis-associated AKI: in cecal ligation-and-puncture (CLP) mice, PERK and eIF2α become phosphorylated and CHOP expression rises sharply. Silencing TIMP2—a biomarker of AKI that also governs cell-cycle arrest—dampens ERS and ameliorates septic injury by restraining the PERK/CHOP pathway (149). Paradoxically, CHOP deletion in lipopolysaccharide-induced AKI aggravates inflammation and tissue injury—a finding likely explained by long-term CHOP deficiency disrupting autophagy, supporting the idea that early-phase UPR signaling can be cytoprotective (150, 151). Other UPR branches likewise modulate AKI. Ischaemia provokes early IRE1α phosphorylation and ATF6 cleavage, hinting that broad UPR engagement constitutes an initial defense against IRI (106, 152). During toxin-induced ERS the IRE1α–XBP1 arm drives secretion of the ribonuclease angiogenin, which curtails protein translation by degrading tRNA and thereby prevents further ER overload; loss of angiogenin makes tubular cells and mice more vulnerable to tunicamycin injury (104, 153, 154). Yet IRE1 signaling can swing both ways. XBP1^+/− mice resist renal ischaemia, apparently because lower XBP1 limits expression of the ubiquitin ligase HRD1; with less HRD1, NRF2 escapes degradation, bolstering antioxidant and anti-apoptotic defenses (155). Tubule-specific XBP1 deletion also lessens septic AKI, whereas sustained XBP1 overexpression induces BiP and CHOP, precipitating progressive AKI—a testament to the damage that follows UPR disequilibrium (156). However, Upregulation of XBP1 in mouse kidney units not only induces the expression of ER luminal Hsp70 (BiP) but also induces CHOP, which is associated with the progression of severe AKI, showing that UPR imbalance may cause irreversible damage (156). Taken together, the impact of UPR activation hinges on the nature and timing of injury, the experimental context and the compensatory networks that are engaged.

##### 2.2.3.2 Adaptive and maladaptive autophagy

Autophagy is a highly conserved cellular mechanism that delivers cellular components to lysosomes for degradation (157). As a key process in cellular quality control, autophagy clears damaged organelles, misfolded or aggregated proteins, and mitigates injury from nutrient deprivation, oxidative stress, and hypoxia, thereby protecting the cell (158, 159). Autophagy involves a series of sequential steps: initiation, autophagosome formation, autophagosome fusion, and degradation (160). Autophagy begins with the emergence of a phagophore, a cup-shaped membrane that progressively elongates to envelop portions of cytoplasm and organelles, sealing off to generate a double-membrane autophagosome. This vesicle subsequently merges with a lysosome to create an autolysosome, whose hydrolytic enzymes dismantle the sequestered cargo. Each phase is orchestrated by dedicated autophagy-related proteins. Among them, microtubule-associated protein 1 light chain 3 (LC3B) is central: activation of autophagy converts cytosolic LC3B-I into the lipidated form LC3B-II, which inserts into autophagosomal membranes and drives later maturation steps (160, 161). Another pivotal factor is p62/SQSTM1, a selective autophagy adaptor that anchors to LC3B and, via ubiquitin tags, shuttles ubiquitinated targets into the forming autophagosome for eventual degradation (162). Recent evidence further indicates that, in sodium arsenite-induced AKI, activation of the YAP1-Nrf2-p62 axis promotes p62 accumulation and autophagic flux blockade in tubular epithelial cells, highlighting impaired autophagic flux itself as a key pathogenic event in AKI progression (163).

Recent studies have shown that autophagy interacts with ERS, and both share common features in alleviating cellular stress or triggering cell death under unfavorable conditions. On the one hand, ERS serves as an inducer of autophagy. Moderate ERS can initiate autophagy to clear misfolded or unfolded proteins, thereby reducing ERS and promoting cell survival (164). However, under certain pathological conditions, ERS imbalance may impair autophagic function. For instance, ERS has been reported to inhibit autophagy via the activation of IRE1 kinase (165). Conversely, deletion of XBP1 increases the expression of various autophagy-regulating genes, thereby enhancing baseline autophagy levels (166). The different roles of ERS in autophagic flux may be due to variations in the sensitivity of different cell types to ERS or the duration of ERS (acute or chronic).

**Figure 3:**
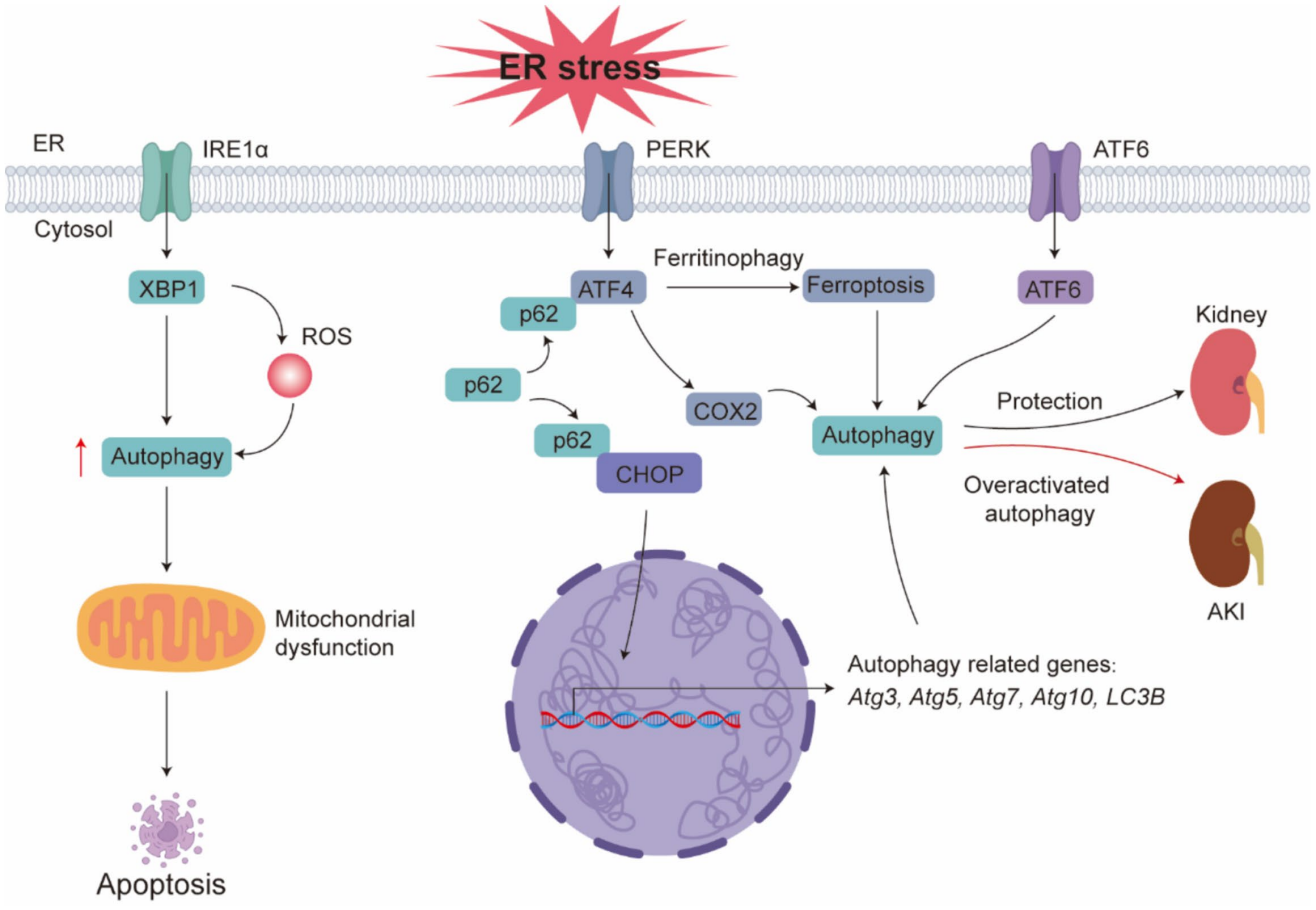
UPR-mediated regulation of autophagy during ER stress. During ER stress, the three UPR branches regulate autophagy in distinct ways. The IRE1 pathway is required for uncontrolled autophagy under ER stress; an IRE1α–ROS positive-feedback loop can sustain high autophagy levels, leading to mitochondrial damage, whereas inhibiting ROS generation suppresses aberrant autophagy and apoptosis. Both PERK and ATF6 can activate autophagy. Under prolonged stress, p62 cooperates with ER-stress signaling and the transcription factors ATF4 and CHOP to activate a set of autophagy genes (Atg3, Atg5, Atg7, Atg10, LC3B), thereby promoting cell survival. The ER stress–eIF2α/ATF4 pathway can also induce autophagy and injury via upregulation of COX2. In addition, the PERK–ATF4 axis mediates ferritinophagy-dependent ferroptosis; inhibition of this pathway or blockade of autophagy significantly attenuates tubular cell injury.

During ERS, the three major UPR pathways regulate autophagy (Figure 3) in different ways (167). The IRE1 pathway is essential for the uncontrolled autophagy that occurs during ERS. In IRE1 knockout mutants, autophagosome formation is not observed when ERS inducers are used (168, 169). Recent studies suggest that IRE1/XBP1-activated autophagy enhances the clearance of misfolded proteins through ER-associated degradation pathways (170). The roles of ATF6 and PERK pathways in regulating autophagy remain controversial. Some studies suggest that autophagy does not rely on ATF6 or PERK signaling: cells with XBP1 knockdown exhibit autophagic cell death, while cells with ATF6 and PERK knockdown display characteristics of programmed cell death (171). Later studies indicated that both the PERK and ATF6 pathways can activate autophagy, either to restore ER homeostasis or to promote programmed cell death. During prolonged stress, p62 interacts with ERS, ATF4, and other transcription factors to activate a set of autophagy-related genes (Atg3, Atg5, Atg7, Atg10, LC3B), promoting cell survival (172). At the same time, the ATF6 pathway is considered essential for autophagy induction mediated by the pestivirus (PPRV) (173).

Studies have shown that the ERS-eIF2α/ATF4 pathway can induce renal autophagy and damage via the upregulation of COX2 in cadmium-induced kidney injury (174). ERS/ATF4 activation increases COX2 expression, further promoting kidney autophagy and damage in lupus nephritis (175). Evidence from cell and animal studies indicates that ER-stress-driven autophagy acts as an adaptive brake during AKI. In cultured proximal-tubule cells, ERS itself is sufficient to spark autophagosome formation (176). Concordantly, renal ischaemia–reperfusion in GFP-LC3 transgenic mice produces abundant GFP-LC3-II puncta within proximal tubules, signaling robust autophagy; if Atg5 is deleted, those mice display more tubular apoptosis, higher serum urea/creatinine and larger p62−/ubiquitin-positive inclusions, underscoring the cytoprotective role of autophagy in this setting (177). A similar pattern emerges with the nephrotoxin cyclosporine. In human tubular cultures, cyclosporine activates the unfolded-protein response (UPR) together with autophagy; dampening ERS with Salubrinal suppresses autophagy, whereas silencing beclin-1 amplifies cell death. *In vivo*, cyclosporine-treated rats show stronger UPR markers and LC3 staining in injured tubules, implying that cyclosporine triggers autophagy through ERS and that this response limits injury (178). Tunicamycin provides another illustration. In LLCPK1 tubule cells, tunicamycin elicits UPR and autophagic flux; chemical inhibitors or beclin-1/Atg5 knock-down block this flux and heighten caspase activity and apoptosis. Tunicamycin-treated mice exhibit more LC3-II puncta in renal tissue, and GFP-LC3 mice reveal numerous fluorescent puncta plus autophagosomes on electron microscopy—often alongside chromatin condensation—showing that autophagy and apoptosis can co-occur in the same damaged tubule cell (179, 180). Death-associated protein kinase (DAPK) sits at the crossroads of these pathways: Dapk-null mice are shielded from tunicamycin nephrotoxicity, indicating that DAPK relays ER-stress signals to both caspases and the autophagy machinery, ultimately tipping the balance toward cell death when the stress is overwhelming (180). When ERS escalates beyond adaptive levels, autophagy may shift from a cytoprotective to a pro-injury modality. In Cd^2+^ toxicity models, early exposure elevates LC3-II and reduces p62, indicating accelerated autophagic flux; however, after 3 hours, concurrent accumulation of LC3-II and p62 reveals impaired autophagosome and lysosome fusion, culminating in apoptosis (181). Moreover, the PERK ATF4 axis mediates ferritinophagy-dependent ferroptosis (a selective form of autophagy in which ferritin is degraded via the autophagolysosomal pathway, releasing free iron and modulating cellular iron homeostasis and oxidative stress), and inhibition of either pathway or general autophagy significantly attenuates Cd^2+^-induced tubular injury (182). Similarly, perfluorooctane sulfonate sustains high autophagy levels via an IRE1α-ROS positive feedback loop, driving mitochondrial damage; L-carnitine intervention reduces ROS, suppresses aberrant autophagy, and prevents apoptosis (183). COMM domain containing 5 (COMMD5) has been shown to preserve autophagosome–lysosome function in cisplatin-injured tubules; COMMD5 loss leads to excessive autophagy with inefficient degradation, resulting in ROS accumulation, mitochondrial failure, and cell death (184). In a prolonged post-ischemic reperfusion model, persistent ERS and elevated autophagy promote interstitial fibrosis; administration of chemical chaperones 3 days post-injury concurrently alleviates ERS, inhibits autophagic activity, and blocks the transition from AKI to CKD (185). A systematic review further indicates that sustained hyperactive autophagy induces tubular epithelial atrophy and a profibrotic phenotype, accelerating maladaptive repair (186). Together, these findings delineate a dynamic balance within the ERS and autophagy axis. Under moderate ERS, autophagy is protective, but once stress exceeds a threshold, CHOP-DAPK signaling (a pathway involving the transcription factor CHOP and death-associated protein kinase, regulating ER stress–induced apoptosis), ferritinophagy, and ROS feedback pivot it toward promoting injury.

#### 2.2.4 Role of endoplasmic reticulum stress in distinct AKI subtypes

##### 2.2.4.1 The role of ERS in renal ischemia–reperfusion injury

IRI frequently occurs in the settings of infection, trauma, major cardiovascular surgery, and renal transplantation (187). Renal IRI is a principal cause of clinical AKI, with persistently high rates of morbidity and mortality (188). Recent studies have underscored the decisive involvement of ERS in IRI pathogenesis. Moderate ERS exerts cytoprotective effects: activation of the UPR by tunicamycin, thapsigargin, or A23187 mitigates IR-induced renal damage (189). Likewise, ERS-triggered autophagy alleviates IRI (179), and pharmacological UPR induction by bolstering autophagic clearance of misfolded proteins and countering oxidative stress and chemical hypoxia—has emerged as a novel therapeutic approach in renal IRI (189). Conversely, excessive or prolonged ERS accelerates IRI progression (190). In murine IRI models, ERS markers GRP78 and CHOP are markedly upregulated, and pharmacologic ERS inhibition reduces tubular apoptosis and improves renal histology (191). Similarly, enhanced expression of GRP78, PERK, ATF4, and XBP1 exacerbates renal dysfunction during IRI (192). The mitochondria-associated ER membrane (MAM) complex is critical for transducing ERS signals to mitochondria and maintaining mitochondrial quality control, both essential for limiting IRI (193). Central to this ER–mitochondrial crosstalk is the IRE1/XBP1 axis: XBP1 activation aggravates tissue injury and mitochondrial fragmentation, whereas XBP1 inhibition suppresses caspase-1-mediated mitochondrial damage, reduces ROS generation, and significantly improves renal function (194). Moreover, ERS-induced NRF2/HO-1 signaling also contributes to IRI pathobiology. Ischemia reperfusion suppresses NRF2/HO-1 activity while upregulating GRP78, eIF2α, and CHOP; pharmacological activation of NRF2/HO-1 reverses these changes and attenuates both functional and structural kidney injury (195). Finally, connexin32 promotes IRI-induced AKI via the ROS/ERS/apoptosis cascade, whereas connexin32 deletion reduces ROS levels, inhibits ERS, and confers renal protection (196).

##### 2.2.4.2 The role of ERS in sepsis-associated AKI

Acute kidney injury is among the most common and severe complications of sepsis, affecting approximately 51% of septic patients (197). Although its exact mechanisms remain incompletely defined, substantial evidence indicates that pathogen-triggered ERS in multiple cell types modulates the inflammatory response during sepsis (198). During sepsis, ERS pathways are robustly activated and closely linked to the onset of sepsis-associated AKI (199). In LPS-injected animal models, intense ERS in the kidney is evidenced by activation of the ATF4/CHOP/caspase-12 axis and upregulation of GRP78 (200). Further studies implicate the IRE1α–JNK pathway in mediating ERS-driven apoptosis in LPS-induced AKI, suggesting that ERS-dependent cell death contributes to septic kidney injury (201). As a key ERS regulator, XBP1 is markedly elevated in the kidneys of LPS-treated mice. Tubule-specific overexpression of XBP1 induces pronounced tubular dilation, vacuolization, and elevated markers of injury (Kim1, neutrophil gelatinase-associated lipocalin (NGAL)) and inflammation (IL-6, TLR4), culminating in worsened renal function and 50% mortality within 5 days. Conversely, XBP1 knockdown markedly reduces NGAL expression, CHOP induction, serum creatinine levels, and TLR4 expression (156). Notably, further LPS challenge in XBP1-overexpressing mice produces only modest additional increases in NGAL and CHOP compared with LPS alone, indicating that amplified XBP1 signaling selectively drives the inflammatory and injurious aspects of septic AKI (156).

##### 2.2.4.3 The role of ERS in drug-induced AKI

In cisplatin-induced AKI, a dynamic interplay also exists between ROS and ERS. In mouse models, salubrinal-mediated enhancement of eIF2α phosphorylation further aggravated ATF4–CHOP signaling, caspase-12/9/3 cleavage, and renal lipid peroxidation, whereas N-acetylcysteine concomitantly improved renal function and reversed these changes, suggesting that cisplatin-induced ROS accumulation can drive ERS from an early adaptive unfolded protein response toward a pro-apoptotic program (112, 202). However, in HK-2 cells, PERK knockdown further increased cisplatin-induced ROS generation, whereas Sal003 enhanced eIF2α phosphorylation, upregulated ATF4 and HO-1, and attenuated oxidative injury, indicating that the PERK–eIF2α–ATF4 axis retains a certain antioxidative buffering capacity during the early phase of injury (203). In addition, in CP-AKI mice, cisplatin was associated with increased mitochondrial ROS and activation of the IRE1 and PERK pathways. Another mouse study further showed that cisplatin promoted ROS generation at the ER surface via CYP2E1 and accelerated the autophagic degradation of Prx I, while Prx I deficiency further amplified oxidative stress in renal tissue, indicating that cisplatin-related ROS sources are not restricted to mitochondria and that the ER itself may also serve as an important platform for amplifying oxidative injury (204, 205).

Contrast-induced AKI exhibits a similarly prominent ROS–ERS coupling pattern. In NRK-52E cells, urografin induced apoptosis in a time- and dose-dependent manner and activated an ERS response characterized by increased GRP78/GRP94 expression and PERK–eIF2α phosphorylation; notably, salubrinal alleviated, whereas GRP78 siRNA aggravated, cell apoptosis (206). However, with sustained stimulation, urografin further enhanced ATF-6- and IRE1–JNK–CHOP-associated pro-apoptotic signaling in rat renal tissue and NRK-52E cells, accompanied by increased Bax, caspase-12, and kim-1 expression; inhibition of caspase-12 partially reversed apoptosis (207). The low-osmolar contrast agent iopromide showed a similar mechanism. In NRK-52E cells, it induced ROS accumulation within 4 h and upregulated GRP78 and CHOP, whereas N-acetylcysteine first suppressed ROS and subsequently attenuated ERS and apoptosis (8). In HK-2 cells, iopromide also activated IRE1α-, eIF2α-, and JNK-related stress pathways, leading to CHOP upregulation, Bax/Bcl-2 imbalance, and caspase-3 cleavage. Both salvianolic acid B and 4-PBA concomitantly suppressed ROS, ERS, and apoptosis, and salvianolic acid B likewise reduced ROS and cell death in a tunicamycin-induced ERS model, suggesting that ERS is not merely a downstream response to oxidative injury but may also feedback to amplify its cytotoxic effects (208). Similarly, in a rat CI-AKI model and iohexol-treated HK-2 cells, contrast media simultaneously induced redox imbalance and ERS activation, as reflected by reduced GSH, increased MDA, and enhancement of the PERK–CHOP–caspase-12 axis. Apelin-13 concomitantly alleviated ROS accumulation, ERS, and apoptosis, whereas tunicamycin weakened its protective effects, further supporting the notion that ERS contributes to sustaining subsequent oxidative stress and cell death (209).

#### 2.2.5 Epigenetic regulation of endoplasmic reticulum stress in AKI

Recent experimental studies have demonstrated that epigenetic mechanisms coordinately modulate both oxidative stress and ERS, profoundly shaping the onset and progression of AKI. In a rat model of I/R, Jiang et al. showed that the methionine-cycle modulator IFC-305 effectively reversed hypermethylation of the cystathionine *γ*-lyase promoter, restored endogenous H₂S synthesis, and reduced levels of superoxide anion and malondialdehyde (MDA), thereby alleviating oxidative damage. Concomitantly, expression of canonical ERS markers GRP78, CHOP, and phosphorylated eIF2α was downregulated, and both tubular necrosis and serum creatinine levels were significantly improved (210). These findings suggest that targeted modulation of gene-specific methylation can synchronously attenuate both oxidative and protein-folding stress. In both a rat I/R model and hypoxia reoxygenation treated HK-2 cells, small nucleolar RNA host gene 14 (lncRNA SNHG14) expression was markedly upregulated. Its overactivation induced sharp intracellular Ca^2+^ fluctuations, excessive ROS production, and elevated GRP78 and CHOP levels. Knockdown of SNHG14 or overexpression of its target, miR-124-3p, not only suppressed oxidative stress but also dampened ERS signaling, thereby mitigating tubular structural injury (211). These results reveal that lncRNA–miRNA networks set the threshold for ERS and ROS burden, decisively influencing cell fate decisions in the early phase of AKI. MicroRNAs themselves can act as precise regulators of the oxidative ERS axis by directly targeting key UPR effectors. MiR-214 is rapidly upregulated following renal I/R; its overexpression targets the transcription factor ATF4, suppressing the ATF4–CHOP axis and GRP78 expression, while concurrently reducing ROS levels and apoptosis to ameliorate tissue injury (212, 213). Collectively, these epigenetic modifications of ERS pathways offer a nuanced layer of control over AKI pathophysiology and suggest novel targets for therapeutic intervention.

#### 2.2.6 ERS-programmed cell-death crosstalk dictates proximal-tubule fate after AKI

Recent multi-omics and single-cell studies have converged on a model in which persistently stressed proximal tubular (PT) epithelial cells survive long-term after injury and drive fibrotic progression, representing the central pathological nexus of maladaptive AKI repair. Kirita et al. first identified a “failed-repair” PT subpopulation via time-course snRNA-seq; these cells maintain chronic UPR signaling (e.g., Atf4, Ddit3) and secrete a repertoire of profibrotic mediators (214). In a ferroptosis-driven AKI model, Ide et al. observed that lipid peroxidation–induced ferroptotic death promotes the accumulation of damage-associated PT (DA-PT) cells, and that iron chelation markedly reduces DA-PT burden, inflammation, and fibrosis (215). Single-cell analyses by Balzer et al. revealed significant enrichment of ERS alongside pyroptosis and ferroptosis pathways in fibrosis-prone PT subsets; pharmacological blockade of these death programs shifts repair toward adaptive outcomes (216). Persistent ERS in PT cells appears to bias cell fate toward a coupled apoptotic–ferroptotic axis, in which CHOP-associated death signaling and IRE1α–JNK-linked ferroptotic stress converge to deplete severely injured tubules while promoting the emergence of pro-inflammatory failed-repair states (215, 217, 218). This is particularly relevant to proximal-tubule fate, because ferroptotic stress not only drives PT cell loss but also promotes the accumulation of inflammatory damage-associated PT cells, thereby linking lethal injury to maladaptive repair and subsequent fibrosis (215). By contrast, pyroptosis seems to be most prominent in inflammatory AKI contexts, where ERS-driven mitochondrial ROS can engage the TXNIP–NLRP3 pathway and amplify tubular inflammation rather than define the universal dominant fate program across AKI etiologies (219). Integrating snRNA-seq with snATAC-seq lineage tracing, Gerhardt et al. demonstrated that SOX9^+^VCAM1^+^ PT cells retain open chromatin at ATF4/CREB3L2 binding sites, sustaining chemokine expression and thereby amplifying profibrotic immune circuits (220). The latest spatiotemporal single-cell atlas further indicates that a ROS-high PT population activates ER–nuclear signaling and senescence programs prior to overt fibrosis, laying the groundwork for AKI to CKD transition (221). Collectively, these data position chronically UPR-active PT cells as the pathological core of maladaptive AKI repair and highlight ERS–cell-death crosstalk pathways as prime targets for therapeutic intervention.

In sum, AKI arises from tight crosstalk between oxidative stress and ER stress. ROS rewire the three branches of the UPR, IRE1α–XBP1, PERK–eIF2α–ATF4–CHOP, and ATF6, while signaling at mitochondria ER contact sites, Ca^2^, and translational arrest further amplify ROS and steer cell fate. Importantly, ROS are both an inducer and a product of ERS, creating a feed-forward circuit in which sustained UPR signaling further enhances mitochondrial ROS generation, lipid peroxidation, and inflammasome activation (218, 219). In ischemic and nephrotoxic AKI, this circuit preferentially feeds into apoptosis and ferroptosis, whereas in septic AKI it can be redirected toward pyroptotic signaling through STING-dependent ERS, mitochondrial ROS overproduction, and TXNIP–NLRP3 activation (218, 219). Transient ER stress and autophagy are adaptive and cytoprotective; sustained activation drives CHOP and DAPK dependent programs, links to ferroptosis and pyroptosis, and culminates in apoptosis, determining whether proximal tubules recover or progress to maladaptive fibrosis. The strength of this axis varies with sex and age, exemplified by estrogen–SIRT3 signaling and NRF2 capacity, and is reset by epigenetic control through DNA methylation and lncRNA or miRNA networks across ischemia–reperfusion and sepsis.

#### 2.2.7 Sex- and age-dependent modulation of oxidative stress and endoplasmic reticulum stress in AKI

In AKI, sex and age can alter the capacity of tubular epithelial cells to cope with oxidative stress and endoplasmic reticulum stress. In a mouse tunicamycin-induced ER stress AKI model, male mice exhibited more pronounced renal dysfunction and proximal tubular injury than female mice, accompanied by stronger induction of BiP, XBP1, and apoptosis-related signaling; after testosterone supplementation in female mice, both the renal injury phenotype and ER stress markers shifted toward the male pattern (116). In a mouse ischemia–reperfusion injury model, castration attenuated the inflammatory response in male mice, whereas ovariectomy aggravated tubular injury and increased the expression of TNF-*α*, MCP-1, IFN-*γ*, and CCL17 in female mice; these changes were reversed by estrogen replacement, indicating that sex hormones influence the extent to which cellular injury expands to the tissue level through modulation of the inflammatory microenvironment (222). Mechanistic studies further showed that basal renal Sirt3 expression is lower in male mice than in female mice, and that increasing Sirt3 alleviates ischemic AKI, whereas Sirt3 deficiency amplifies injury, supporting Sirt3 as an important determinant of sex-related differences (118). After upregulation by E2 through the estrogen receptor, SIRT3 regulates OPA1 processing via deacetylation of YME1L1, thereby shifting mitochondrial dynamics toward fusion, reducing mitochondrial ROS production, and attenuating hypoxia-reoxygenation-induced ER stress; conversely, SIRT3 knockdown enhances oxidative stress, promotes mitochondrial fission, aggravates ER stress, and abolishes the protective effect of E2 (117). This finding is consistent with previous renal IRI studies showing that Sirt3 is downregulated under both *in vivo* and *in vitro* IRI conditions, whereas its overexpression reduces ROS, preserves mitochondrial membrane potential, promotes mitochondrial fusion through the ERK-OPA1 pathway, and decreases apoptosis of tubular epithelial cells (223); SIRT3 can also buffer IRI-induced injury by regulating DRP1-related mitophagy (224).

Aged rats exhibit higher levels of BUN, serum creatinine, and renal 8-OHdG after IRI, together with reduced plasma antioxidant capacity, suggesting that aging primarily impairs the antioxidant buffering capacity during reperfusion (225). Another long-term follow-up study in rats further showed that renal MDA and 8-OHdG were more markedly increased in the aged IRI group and were accompanied by more severe inflammatory responses and interstitial fibrosis, indicating that oxidative injury not only contributes to acute renal damage but also continues to affect the subsequent repair process (226). More importantly, aged mice are more susceptible to tunicamycin-induced ER stress-related renal injury, as reflected by loss of XBP1 splicing and attenuation of late PERK-eIF2α phosphorylation; exogenous antioxidant intervention restored IRE1-XBP1 signaling and alleviated renal injury, indicating that preexisting oxidative stress in the aged kidney can directly impair the adaptive branch of the UPR and render ER stress more likely to shift from a protective response to a deleterious one (90). Recent studies on the recovery phase after renal IRI in aged mice further showed that NRF2 expression is downregulated, with increased MDA levels and aggravated fibrosis after IRI, whereas Nrf2 deficiency further exacerbates oxidative stress and mitochondrial OXPHOS dysfunction, suggesting that age-related redox imbalance may prolong the post-AKI stress state and increase the likelihood of progression toward chronic kidney injury (227).

### 2.3 Therapeutic modulation of oxidative and ERS

ERS, particularly its associated UPR, has been recognized as a potential cause and consequence of AKI. Given the established link between oxidative stress, ERS, and AKI, modulating oxidative stress to alleviate ERS presents a promising strategy for ameliorating AKI (see Table 1).

**Table 1:** Application of agents that alleviate oxidative stress and endoplasmic reticulum stress in the treatment of AKI.

Therapeutic agent	Main molecular target/pathway	AKI model (inducer)	Experimental model	Study type	Key effects	Reference
EGCG	ROS-driven ER-stress signaling (reduced GRP78, CHOP and downstream apoptosis)	Rhabdomyolysis (glycerol)	Wistar rats HEK-293 cells	Pre-clinical	Reduces ER stress and oxidative stress elevated by glycerol treatment; reverses tubular apoptosis rate in H₂O₂-treated HEK-293 cells.	(235)
CORM2	ROS–Fyn kinase–dependent ER-stress cascade	Endotoxin (LPS)	C57BL/6 J mice	Pre-clinical	Inhibits oxidative stress, Fyn activation, ER stress, and apoptosis in AKI mice and H₂O₂-stimulated proximal tubular epithelial cells.	(237)
JQ1	Brd4/FoxO4 oxidative-stress axis	Ischaemia-reperfusion	C57 mice	Pre-clinical	Prevents FoxO4-dependent ROS production via the PI3K/AKT pathway, thereby blocking renal apoptosis and ER stress protein expression.	(239)
Dexmedetomidine	Overall ER-stress suppression, lower GRP78, CHOP; mitigated oxidative stress	Myocardial I/R–induced AKI	Sprague–Dawley rats	Clinical	Reduces malondialdehyde levels and increases superoxide dismutase levels during reperfusion; significantly decreases ATF-6 expression and myocardial injury markers cTnI and CK-MB in MI/RI rats, thereby alleviating MI/R-induced AKI.	(242)
Apelin-13	Inhibition of ER-stress markers GRP78, CHOP, caspase-12; relief of ROS load	Contrast-induced (iohexol)	Sprague–Dawley rats	Pre-clinical	Ameliorates endoplasmic reticulum stress, reactive oxygen species, and apoptosis-related protein expression in contrast media-treated cells and rat kidney tissues.	(209)
Maresin-1	Activation of AMPK/SIRT3 with concurrent down-regulation of p-PERK, GRP78, p-eIF2α, ATF4, CHOP and pyroptosis proteins	Sepsis (CLP)	BALB/C mice	Pre-clinical	Activates the AMPK/SIRT3 signaling pathway; improves endoplasmic reticulum stress-related markers p-PERK/PERK, GRP78, p-EIF2α/EIF2α, ATF4, and CHOP; attenuates renal injury in septic mice.	(245)
Xuebijing (XBJ) injection	Modulation of TLR4/MyD88/NF-κB axis; mitigation of mitochondrial dysfunction and ER-stress	Sepsis (CLP)	C57BL/6 mice HK-2 cell	Pre-clinical	Reduces ROS production and inhibits ER stress under LPS stimulation, thereby treating sepsis-induced acute kidney injury.	(247)
Resveratrol	Activates Nrf2, which in turn decreases ROS and lowers p-IRE1, GRP78, CHOP and NF-κB phosphorylation	Sepsis (CLP)	C57BL/6 J mice HK-2 cells	Pre-clinical	Less tubular injury and improved survival.	(251)
NaHS	Inhibits the PERK–eIF2α–CHOP axis, thereby reducing cytosolic and mitochondrial ROS	Sepsis (LPS-AKI)	C57BL/6 J mice	Pre-clinical	Restores Bax/Bcl-2 balance and decreases apoptosis.	(252)
MitoQ	Scavenges mitochondrial ROS, leading to lower 4-HNE/8-OHdG and reduced GRP78 and CHOP expression	Ischaemia–reperfusion	Sprague–Dawley rats	Pre-clinical	MRI and histology confirm attenuation of outer-medullary injury.	(254)

EGCG, epigallocatechin gallate; CORM, carbon monoxide-releasing molecule.

#### 2.3.1 Chemical chaperones

Chemical chaperones that alleviate ERS and oxidative stress have demonstrated consistent renoprotective effects across diverse AKI models. In tunicamycin-induced AKI in mice, 4-phenylbutyrate (4-PBA) markedly suppresses GRP78 and CHOP expression, lowers serum creatinine and BUN, and reduces proximal tubular necrosis (228). In ischemia–reperfusion models, 4-PBA attenuates the upregulation of p-eIF2α and GRP78, thereby diminishing epithelial apoptosis and inflammatory responses (229). In cisplatin-induced AKI, 4-PBA inhibits PDK4 to decrease ROS generation, improving renal function without compromising cisplatin’s antitumor efficacy (230). Tauroursodeoxycholic acid (TUDCA) exhibits similar protective properties. In murine renal I/R injury, TUDCA suppresses GRP78/CHOP and significantly reduces epithelial cell apoptosis (231). In rat and HK-2 cell reoxygenation–reperfusion experiments, TUDCA blocks ERS-dependent mitochondrial apoptotic pathways, enhances survival, and ameliorates renal function (232). Ursodeoxycholic acid (UDCA) also merits mention: in an endotoxin-induced sepsis AKI model, UDCA activates the Nrf2/HO-1 axis and inhibits NF-κB, thereby reducing oxidative stress and inflammatory burden and improving serum creatinine and BUN (233). Collectively, these studies indicate that chemical chaperones such as 4-PBA and TUDCA stabilize the protein-folding environment and mitigate oxidative stress under varying etiological conditions, protecting proximal tubules and interrupting AKI progression.

#### 2.3.2 Small molecule interventions beyond chemical chaperones

Regulation of oxidative stress and ERS has been shown to provide effective protection against acute kidney injury. For example, Su et al. demonstrated that knockout of fibronectin A3 can reverse oxidative stress and ERS induced by LPS treatment, protecting HK2 cells from LPS-induced cell damage (234). Chang et al. found that administering epigallocatechin gallate (EGCG) to male Wistar rats significantly reduced ERS and oxidative stress induced by glycerol, thereby reversing the apoptosis rate in H_2_O_2_-treated HEK-293 cells (235). However, preclinical data indicate that escalating the dose of EGCG to 750 mg·kg^−1^ induces moderate to severe hepatic necrosis accompanied by marked oxidative damage. Moreover, in diabetic murine models, the same dosage paradoxically exacerbates renal toxicity. These findings underscore the necessity for careful dose optimization in clinical settings and heightened vigilance in populations with underlying metabolic disturbances (236). Carbon monoxide (CO) has been shown to alleviate oxidative stress and ERS in various cells. Treatment of C57BL/6 J mice with 30 mg/kg CORM2 effectively suppressed oxidative stress, Fyn activation, ERS, and cell apoptosis in AKI (237). Pre-treatment with CORM2 also demonstrated inhibition of oxidative stress, Fyn activation, ERS, and apoptosis in proximal renal tubular epithelial cells stimulated with H_2_O_2_ (237). However, subsequent investigations have revealed that CORM-2 liberates only trace amounts of CO, with its antibacterial and cytotoxic activities largely attributable to intracellular ruthenium accumulation. The resulting DNA damage and decreased cell viability underscore metal residue as a critical barrier to its further development (238). The selective BRD4 inhibitor JQ1, blocks FoxO4-dependent ROS production through the PI3K/AKT pathway, thereby preventing kidney apoptosis and ERS protein expression, suggesting BRD4 as a potential therapeutic target for renal I/R injury (239). Notably, sustained BRD4 inhibition in transgenic mice induces multi-organ toxicity, manifesting as depletion of small intestinal stem cells, skin hyperplasia, and weight loss (240). Moreover, JQ1’s inhibition of BRDT produces reversible male infertility, underscoring its off-target reproductive toxicity risks (241).

Dexmedetomidine, widely used to alleviate myocardial ischemia–reperfusion-induced acute kidney injury, has been shown to lower malondialdehyde levels during reperfusion, increase SOD content, and significantly reduce ATF-6 expression in MI/RI rats, indicating that it mitigates oxidative stress and ERS, thereby reducing AKI caused by MI/R injury (242). A clinical retrospective study reported that dexmedetomidine induced hypotension or bradycardia in over 70% of critically ill patients within 24 h, with even greater risk observed in the elderly, those with low baseline blood pressure, or patients receiving concomitant vasoactive agents (243). Apelin, an endogenous physiological regulator with antioxidant, anti-inflammatory, and anti-apoptotic properties, was reported by Liu et al. to normalize ERS, ROS, and apoptosis-related protein expression in cells and rat kidney tissues treated with contrast agents (209). Apelin-13 is, however, a short peptide with a plasma half-life of only minutes. Although N-terminal fatty-acid conjugation can extend its half-life to several hours, this modification concurrently increases manufacturing costs and dosing complexity (244). Maresin-1, a lipid mediator that promotes the resolution of inflammation, protects organs from inflammatory damage by facilitating the resolution of acute inflammation. Studies have demonstrated that Maresin-1 activates the AMPK/SIRT3 signaling pathway, improves ERS-related markers such as p-PERK/PERK, GRP78, p-EIF2α/EIF2α, ATF4, and CHOP, and reduces kidney damage in sepsis mice (245). The activation of the AMPK/SIRT3 signaling pathway has been shown to relieve oxidative stress, suggesting that Maresin-1 may alleviate oxidative stress and provide renal protection through this pathway (246). Zhang et al. demonstrated that Xuebijing (XBJ) injection significantly reduces ROS production and inhibits ERS, thereby treating sepsis-induced AKI (247). A surveillance study covering 31,913 patients across 93 hospitals reported an overall adverse reaction incidence of approximately 0.3% for Xuebijing injection, predominantly manifesting as pruritus and erythema, with events linked to rapid infusion rates and infusion device factors (248). The plant extract thymidine quinone (THQ) has antioxidant and anti-inflammatory properties, and studies have shown that intraperitoneal injection of THQ effectively reduces total oxidative status, suppresses ERS, and apoptotic pathways, thus mitigating acute kidney injury (249). Nonetheless, administration of high-dose THQ has been shown to induce DNA strand breaks and necrotic cell death, underscoring the relatively narrow safety window of its injectable formulation (250). Nrf2 activation by resveratrol markedly attenuates oxidative and ERS in both a cecal ligation–puncture rat sepsis model and LPS/tunicamycin–treated HK-2 cells, as evidenced by reductions in DHE and MitoSOX fluorescence and suppression of p-IRE1, GRP78, and CHOP expression (251). Similarly, the H₂S donor NaHS decreases DCFH-DA–detected ROS levels, inhibits the PERK–eIF2α–CHOP axis, and restores the Bax/Bcl-2 balance in LPS-induced AKI mice (252). In a warm ischemia–reperfusion rat model, melatonin reduces MDA and lactate dehydrogenase (LDH) levels, downregulates ERS markers GRP78, XBP1, and CHOP, and improves renal function (253). The mitochondria-targeted antioxidant MitoQ diminishes 4-HNE (4-hydroxynonenal) and 8-OHdG (8-hydroxy-2′-deoxyguanosine) accumulation, suppresses GRP78 and CHOP expression, and, by MRI, has been shown to significantly alleviate outer medullary injury in I/R models (254). Together, these findings underscore the therapeutic potential of concomitantly modulating ROS and ERS to ameliorate AKI. Bardoxolone methyl is a synthetic triterpenoid Nrf2 agonist with potent antioxidant activity. By disrupting the Keap1–Nrf2 interaction, it promotes nuclear accumulation of Nrf2 and induces downstream cytoprotective genes such as HO-1 and NQO1, thereby attenuating reactive oxygen species accumulation and oxidative injury (255). In a murine ischemia–reperfusion AKI model, bardoxolone methyl significantly improved renal function and histopathological injury, while increasing renal expression of Nrf2, PPARγ, and HO-1, indicating that enhancement of endogenous antioxidant defense contributes to its renoprotective effect (256). Similarly, in an aristolochic acid-induced AKI model, bardoxolone methyl reduced blood urea nitrogen and serum creatinine levels, ameliorated tubular necrosis, and upregulated renal Nrf2, HO-1, and NQO1 expression with concomitant suppression of Keap1, further supporting its protective role against nephrotoxic AKI through activation of the antioxidant response pathway (257). In addition, in a rat renal ischemia–reperfusion injury model, bardoxolone methyl decreased urea, creatinine, NGAL, total oxidant status, nitric oxide, and ADMA levels, increased SOD and GSH-Px activities, and upregulated renal Nrf2 and PPARγ expression, suggesting that its anti-oxidative, anti-inflammatory, and anti-apoptotic effects collectively mitigate AKI severity (258).

#### 2.3.3 Clinical interventions targeting oxidative and ERS in AKI

In recent years, although clinical evidence remains limited, strategies aimed at attenuating oxidative stress and thereby mitigating ERS in AKI have shown promising advances. Short-course oral administration of coenzyme Q10 significantly reduced the incidence of contrast-induced AKI and ameliorated elevations in serum creatinine and urinary NGAL in patients (259) in a randomized, placebo-controlled clinical trial (trial registration IRCT20120215009014N414, completed). In parallel, animal studies demonstrated that coenzyme Q10 downregulated GRP78, phosphorylated IRE1α/XBP1s, and CHOP, while inhibiting RIP1–RIP3–MLKL-mediated necroinflammation (260). In renal transplant recipients, melatonin supplementation decreased early post-transplant urinary NGAL, MDA, and 8-OHdG levels, and accelerated recovery of graft function (261) in a double-blind, randomized, placebo-controlled clinical trial (trial registration IRCT201610203812N5, completed); in an ischemia–reperfusion model, melatonin similarly alleviated ERS-dependent apoptosis by suppressing the PERK/eIF2α–ATF4–CHOP axis and restoring Akt/GSK3β signaling (253). N-acetylcysteine lowered the incidence of contrast-induced AKI (262) in a single-center, prospective, randomized, placebo-controlled clinical trial (trial registration not reported), and in diabetic rat models it inhibited GRP78/CHOP and phosphorylated PERK while upregulating Bcl-2, thus attenuating ERS-triggered apoptosis (263). Although a combined vitamin C regimen did not further reduce AKI in the same trial, other nephrotoxicity models indicate that high-dose vitamin C can suppress the PERK–ATF4–CHOP pathway (264). Collectively, these interventions, by reducing reactive oxygen species and modulating ERS, have begun to demonstrate clinical potential. Future studies incorporating simultaneous measurement of ERS markers such as GRP78, CHOP, and XBP1s will be essential to delineate the causal interplay among oxidative stress, ERS, and AKI. Melatonin, an endogenous antioxidant with free radical-scavenging properties, has also been evaluated in clinical AKI-related settings. In a double-blind, randomized, placebo-controlled trial involving 40 renal transplant candidates, melatonin reduced urinary NGAL and multiple oxidative stress markers, with renal injury biomarkers, inflammatory mediators, and oxidative stress indices as study endpoints (261). In addition, a randomized, double-blind, placebo-controlled trial in 40 patients with chronic kidney disease undergoing coronary angiography used contrast-induced AKI as the primary endpoint and showed that melatonin reduced CI-AKI incidence and attenuated NGAL elevation (265). N-acetylcysteine, a classic antioxidant and glutathione precursor, has been more extensively studied as a preventive intervention in AKI. In the large PRESERVE randomized trial, 5,177 high-risk patients undergoing angiography were assigned to oral N-acetylcysteine or placebo, and the primary endpoint was a composite of death, dialysis, or persistent renal function decline at 90 days, with contrast-associated AKI as a secondary endpoint; however, no significant benefit was observed (266). In cardiac surgery, randomized trials have reported inconsistent findings: one placebo-controlled study in 102 patients with chronic kidney disease, using postoperative serum creatinine change as the primary endpoint, found no renoprotective effect (267), whereas another trial in 70 patients undergoing coronary artery bypass grafting reported a reduction in AKI incidence with high-dose intravenous administration (268).

## 3 Conclusion and future directions

AKI is a syndrome with multifactorial etiology, and its incidence continues to rise worldwide. Oxidative stress plays a key role in the onset and progression of AKI. Excessive production of ROS triggers a series of pathological processes, including the activation of ERS. ERS regulates cellular adaptation to oxidative stress and other external pressures through the UPR. However, when ERS is prolonged or excessive, the UPR can lead to cell death and exacerbate kidney damage. The main signaling pathways of UPR, including IRE1α, PERK, and ATF6, regulate cell survival and death through mechanisms including apoptosis and autophagy. During the development of AKI, oxidative stress and ERS pathways intertwine and jointly promote the occurrence and progression of AKI. First, ROS are generated through various pathways, such as mitochondrial dysfunction due to sirtuin deficiency and NADPH oxidase activation, leading to cellular damage and further activation of ERS. Notably, the mitochondria-targeted antioxidants have been shown to efficiently scavenge mtROS and inhibit downstream TGF-β1/NF-κB–mediated ERS signaling, conferring robust renoprotection in AKI models. ERS, through the activation of UPR and key transcription factors such as XBP-1 and CHOP, regulates cellular stress responses and metabolic processes. Prolonged or severe stress shifts this balance, activating both autophagy and apoptosis. Autophagy can help cells adapt to stress by removing damaged organelles and misfolded proteins, but under excessive stress, dysregulated autophagy can accelerate cell death. Additionally, ERS activates apoptotic pathways via UPR signaling, and the upregulation of CHOP can directly induce apoptosis by activating caspase cascades, further aggravating the pathological progression of AKI.

Although considerable progress has been made in elucidating the interaction between oxidative stress and ERS in AKI has been established, further in-depth studies on their precise mechanisms are needed. Specifically, the dual role of UPR in different types of kidney injury—both protective and promoting cell death—requires clarification in various pathological conditions. In addition, although AKI subphenotyping is emerging as a promising framework for precision medicine, direct evidence linking oxidative stress and ERS to specific AKI subtypes remains limited. Defining subtype-specific stress-response patterns and therapeutic vulnerabilities may therefore represent an important direction for future research. Moreover, how oxidative stress and ERS jointly influence specific signaling pathways, such as NF-κB, MAPK, and SIRT1, and how they affect the balance between autophagy and apoptosis, remains an important question to address. Moreover, how oxidative stress and ERS jointly influence specific signaling pathways, such as NF-κB, MAPK, SIRT1, and how they affect the balance between autophagy and apoptosis, remains an important question to address. As the understanding of AKI mechanisms deepens, new therapeutic strategies should focus on improving AKI outcomes by modulating oxidative stress and ERS. For example, antioxidant agents and ERS regulators have shown promising efficacy in experimental studies, helping alleviate kidney injury by relieving oxidative stress and inhibiting ERS. However, the clinical translation of these treatments still faces significant challenges, particularly regarding how to selectively modulate these pathways without interfering with other metabolic processes. Therefore, future research should aim to develop novel targeted drugs that specifically modulate ROS generation and ERS responses, to provide more effective therapeutic options for AKI patients.
